# Prediction of Blast Vibration Velocity of Buried Steel Pipe Based on PSO-LSSVM Model

**DOI:** 10.3390/s24237437

**Published:** 2024-11-21

**Authors:** Hongyu Zhang, Shengwu Tu, Senlin Nie, Weihua Ming

**Affiliations:** 1 Hubei Key Laboratory of Blasting Engineering, Jianghan University, Wuhan 430056, China; 230204030002@hhu.edu.cn (H.Z.); forrester.nie@foxmail.com (S.N.); weihuaming1990@163.com (W.M.); 2 State Key Laboratory of Precision Blasting, Jianghan University, Wuhan 430056, China; 3 College of Civil and Transportation Engineering, Hohai University, Nanjing 210000, China

**Keywords:** blast load, buried pipeline, vibration velocity prediction, least squares support vector machine, particle swarm optimization

## Abstract

In order to ensure the safe operation of adjacent buried pipelines under blast vibration, it is of great practical engineering significance to accurately predict the peak vibration velocity ofburied pipelines under blasting loads. Relying on the test results of the buried steel pipe blast model test, a sensitivity analysis of relevant influencing factors was carried out by using the gray correlation analysis method. A least squares support vector machine (LS-SVM) model was established to predict the peak vibration velocity of the pipeline and determine the best parameter combination in the LS-SVM model through a local particle swarm optimization (PSO), and the results of the PSO-LSSVM model were predicted. These were compared with BP neural network model and Sa’s empirical formula. The results show that the fitting correlation coefficient (R2), root mean square error (RMSE), average relative error (MRE), and Nash coefficient (NSE) of the PSO-LSSVM model for the prediction of pipeline peak vibration velocity are 91.51%, 2.95%, 8.69%, and 99.03%, showing that the PSO-LSSVM model has a higher prediction accuracy and better generalization ability, which provides a new idea for the vibration velocity prediction of buried pipelines under complex blasting conditions.

## 1. Introduction

The main factor that threatens the safety of buried pipelines from blasting operations is a blast’s seismic wave, which will cause the deformation and vibration of pipelines, resulting in different degrees of damage to the pipe’s structure; so, accurately predicting the vibration effect generated in the blasting process and optimizing the blasting design parameters are the main methods to reduce the damage of the blast vibration effect on buried pipelines [1,2]. At present, the main methods used to predict the vibration velocity of blasting are the empirical formula method [3,4,5,6,7], the BP neural network and its improved algorithm [8,9,10,11], numerical simulation [12,13,14], etc. Sa’s existing empirical formula or the improved formula based on it has few factors to consider, and the prediction error under the influence of multiple factors is significant, so it can only be adapted to specific blasting projects. The BP neural network needs a large number of training samples to improve the model’s prediction accuracy, which does not meet the actual needs of engineering. Numerical simulation methods often require strong numerical computing skills, and can usually only obtain specific solutions under certain conditions, which are not universal. Therefore, it is important to explore a method that considers more factors and exhibits a higher prediction accuracy, which provides a new direction for the prediction of blast vibration effects under the influence of complex factors, and is of great significance in seismic disaster mitigation for buried pipelines.

With the development of computer network technology and machine learning methods, a large number of scientific methods with strong nonlinear processing capabilities and real-time learning have gradually emerged. As an emerging machine learning algorithm, support vector machine (SVM) has a strong optimization ability and can solve practical problems such as a small number of samples, nonlinearity, and many influencing parameters in blasting engineering [15]. Wei et al. [16], based on the nonlinear regression theory of support vector machines, proposed an SVM model for predicting blast vibration velocity, offering a new approach for blast vibration prediction. Peng Fuhua et al. [17] proposed an SVM model using peak vibration velocity prediction, suggesting that it is feasible to use the SVM model to predict blast vibration peaks. Zhang Pengfei et al. [18] conducted research on blast vibration prediction in open-pit coal mines and proposed a gray relational analysis-based feature selection model using an integrated particle swarm optimization—support vector machine (GRA-EPSO-SVM) algorithm, which improved the accuracy of peak blast vibration prediction in open-pit mines. Ke et al. [15] mixed the neural network and support vector regression models to form a hybrid intelligent model, which improved the prediction accuracy of ground motion intensity. Yue Zhongwen [19] et al. proposed to optimize the SVM model by combining principal component analysis and a genetic algorithm. Their research results show that the convergence speed and prediction accuracy of the model were improved. Xu et al. [20] combined principal component analysis (PCA) and support vector machine (SVM) to simplify the input parameters of their model, which was used to adjust mining blast parameters. Compared to traditional prediction models, the PCA-SVM model demonstrated higher accuracy in predicting blast vibrations in mining. He Li [21] et al. established a least squares support vector machine (LS-SVM) model to predict the vibration speed of mine blasting. The results show that the PSO-LSSVM model has a higher prediction accuracy and can be used to predict the PPV of mine blasting under the influence of multiple factors. The least squares support vector machine (LS-SVM) model changes the inequality constraints in the SVM model into equality constraints, which can greatly reduce the difficulty and complexity of calculations. Particle swarm optimization (PSO), as a new type of global search algorithm, has the characteristics of few parameters, fast convergence speed, and high prediction accuracy, and has been widely used in parameter optimization. By combining a particle swarm optimization (PSO) algorithm with machine learning models such as SVM and RF, the accuracy and reliability of vibration prediction models can be further improved [22].

Based on the results of the underground pipeline blast model test conducted by the Hubei Provincial Key Laboratory of Blasting Engineering [23,24,25], this paper established a buried steel pipe blast vibration velocity prediction model based on PSO-LSSVM. The gray correlation analysis method was used to conduct sensitivity analysis on various factors influencing the measured buried steel pipes, and the primary and secondary relationships between various influencing factors were determined. The PSO algorithm’s local optimization was used to determine the best combination of regularization parameters and kernel function width coefficients in the LS-SVM model, and the experimental and numerical simulation data were combined to predict the blast vibration speed of buried steel pipes. By comparing and analyzing the prediction results of the PSO-LSSVM model, the BP neural network model, and the Sadowsky formula, the results show that the prediction accuracy of the PSO-LSSVM model is higher. These relevant research results can provide new ideas for predicting the vibration speed of buried pipelines adjacent to blasting projects.

## 2. Establishment of LS-SVM Prediction Model Based on PSO

### 2.1. Basic Principles of PSO-LSSVM

Support vector machine (SVM) is a type of generalized linear classifier that performs binary classification of data. Its decision boundary is the maximum margin hyperplane that solves the learning sample. The LS-SVM algorithm is an optimization of the standard SVM algorithm. The main optimization feature is adding the equality constraints, which turns solving the inequality constraints into solving linear equations, reducing the complexity of the algorithm [26]. The final optimization function of LS-SVM is

(1) $$f(x)=\sum_{i=1}^{n}\xi_{i}K\left(x,x_{i}\right)+b$$

In the formula: $K\left(x,x_{i}\right)$ is the kernel function; b is the bias constant; and $\xi_{i}$ is the Lagrange multiplier.

The kernel function in this article is the Gaussian kernel function, and the expression is

(2) $$K\left(X_{i},X_{j}\right)=\exp\left(-\frac{{\left\|X_{i}-X_{j}\right\|}^{2}}{2\sigma^{2}}\right)$$

In the formula: σ is the kernel width of the Gaussian kernel function; $\left\|X_{i}-X_{j}\right\|$ is the module of the vector; and $X_{i},X_{j}$ are the two sample sets.

Researchers select the regularization parameter *γ* and kernel function width coefficient *σ* of the LS-SVM model by experience, but the resulting model is often not optimal. Therefore, the particle swarm algorithm (PSO) is used to iteratively optimize these two parameters of the LS-SVM model to improve the prediction accuracy and convergence speed of the model. The particle swarm algorithm is an evolutionary calculation algorithm proposed by Kennedy and Eberhart [27]. The algorithm is inspired by the social behavior of organisms, such as bird gatherings and groups of fish. The algorithm consists of a group of particles that find the best position based on its best solution, including the best individual position ($p_{best}$) and the best global position ($g_{best}$). In PSO, the formula of the movement process of particles according to their position and velocity is

(3) $$V_{\mathrm{new}\;}=w\times v+C_{1}\cdot r_{1}\left(p_{\mathrm{best}\;}-X\right)+C_{2}\cdot r_{2}\left(g_{\mathrm{best}\;}-X\right)$$

(4) $$X_{\mathrm{new}\;}=X+V_{\mathrm{new}\;}$$

In the formula: $C_{1}$ and $C_{2}$ are learning factors; V and X represent the speed and position of the current particle; $V_{new}$ and $X_{new}$ are the new speed and new position of the particle; w is the inertial weight; and $r_{1}$ and $r_{2}$ are the random numbers in [0, 1].

### 2.2. The Process of the PSO-LSSVM Model

**Figure 1:**
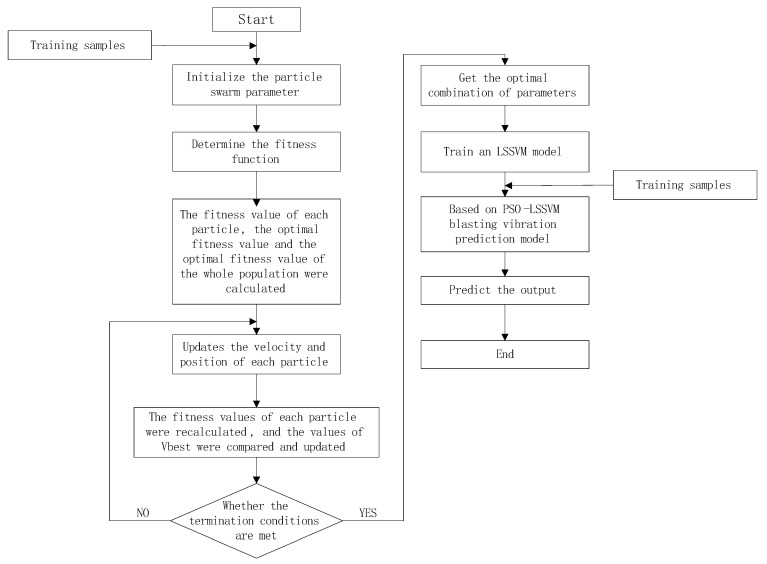
Processing flow of PSO-LSSVM model.

The LSSVM model is used to establish the nonlinear relationship between the peak blast vibration speed and its influencing factors to predict the peak blast vibration speed. The PSO algorithm is used to find the best combination of LSSVM key parameters *γ* and *σ*, and the peak blast vibration based on PSO-LSSVM is constructed. The specific process of the speed prediction model is shown in Figure 1.

## 3. Gray Correlation Analysis of Factors Affecting Vibration Velocity of Buried Pipelines

### 3.1. Model Test Overview

**Figure 2:**
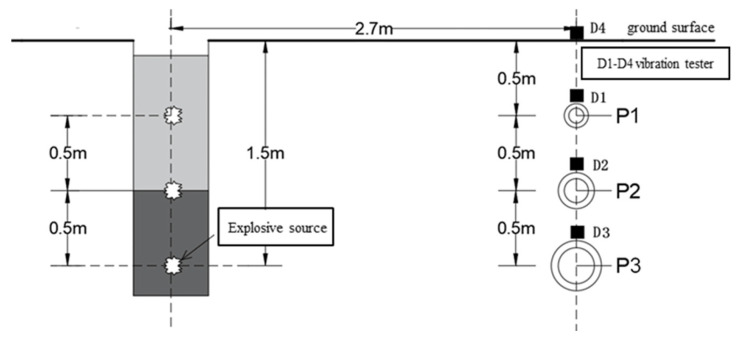
Model test layout diagram.

**Figure 3:**
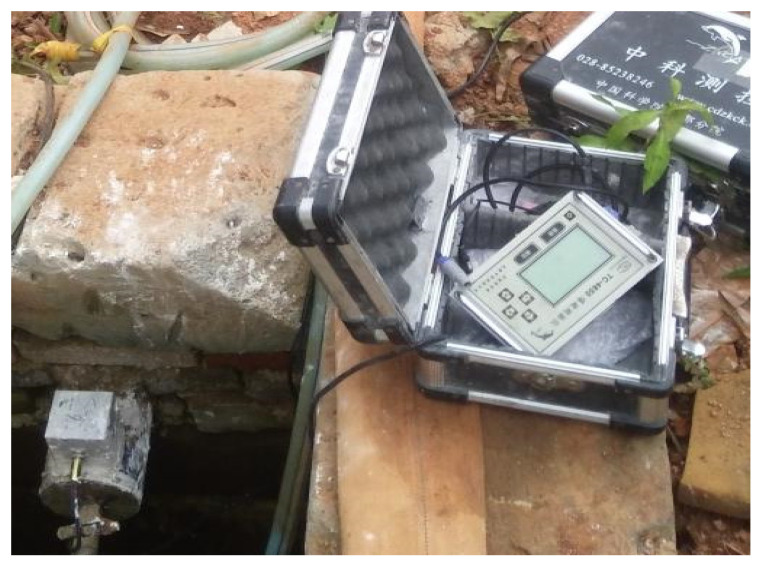
Installation diagram of blast vibration meter.

The test site is located in the open space of Hubei Provincial Key Laboratory of Explosive Engineering. The soil medium at the site is mainly yellow clay. The test object uses 20# seamless carbon steel pipes commonly used for oil and gas transportation in urban construction. The pipe laying method is direct burial. Large excavation machinery was used to excavate the pipe trench, and the site soil was backfilled after the completion of pipe laying. In the blast test, 2# Rock Emulsion Explosive was used, made into a spherical charge bag, adopting a coupled charging method. In the experiment, the TC-4850 blast vibration meter was used to detect the blast vibration speed of each buried pipeline and the ground. The test arrangement is shown in Figure 2, and the blast vibration meter arrangement is shown in Figure 3.

The parameters changed in the experiment include pipeline internal pressure, explosion center distance, explosion source burial depth, and charge amount. The control variable method was used to design the experiment, and the experimental plan was further optimized according to the orthogonal method. The test parameters and control variable ranges are shown in Table 1.

The test uses three 20# seamless steel pipes with different nominal diameters as the research objects. The geometric parameters and material mechanical property parameters of the steel pipes are shown in Table 2 and Table 3.

### 3.2. Gray Correlation Analysis of Factors Affecting Pipeline Vibration Speed

The basic idea of the gray correlation analysis method is to judge whether the connection is close based on the similarity of the geometric shapes of the sequence curves. The closer the curves are to one another, the greater the gray correlation between the corresponding sequences, and vice versa. The gray correlation analysis method is used to calculate the gray correlation between the system’s characteristic variable data sequences, establish a gray correlation matrix, use the principle of advantage analysis to obtain the order of each influencing factor, and finally determine the main influencing factors.

The general expression of correlation coefficient is

(5) $$\gamma_{i}=\frac{1}{n}\overset{i=1}{\underset{n}{\sum }}\varepsilon_{i}(k)(k=1,2,\cdots ,n)$$

In the formula: $\gamma_{i}$ is the correlation coefficient and $\varepsilon_{i}$ is the correlation coefficient.

In the experiment, 60 data sets suitable for training were obtained; the data sets selected for the model test are shown in Table 4. These include eight characteristic parameters: charge quantity *Q*, explosive burial depth *H_e_*, blast center distance *R*, pipeline wall thickness δ, pipeline burial depth *H*, pipeline diameter *D*, pipeline internal pressure *P*, and pipeline peak vibration velocity V.

Substituting these into Equation (5), the correlation degree of the characteristic parameter influencing factor between each parameter and the peak vibration speed V is obtained, and after sorting, Table 5 is obtained.

It can be seen from Table 5 that the characteristic parameter that has the greatest influence on the peak vibration velocity of the pipeline is the charge quantity Q, followed by the blast center distance R, pipeline wall thickness δ, pipeline internal pressure P, and a smaller influence is exerted by the pipeline diameter D, explosive burial. The depth of the river and the pipeline depth H are close to each other. This paper selects seven parameters as the input variables of the model. In actual engineering blasting, by considering issues such as on-site test costs and the calculation efficiency of the prediction model, the charge quantity Q, blast center distance R, pipe wall thickness δ, and pipe internal pressure can be selected. The five characteristic parameters P and pipe diameter D are used as input variables.

## 4. Application and Analysis of PSO-LSSVM Prediction Model

### 4.1. Model Building

**Figure 4:**
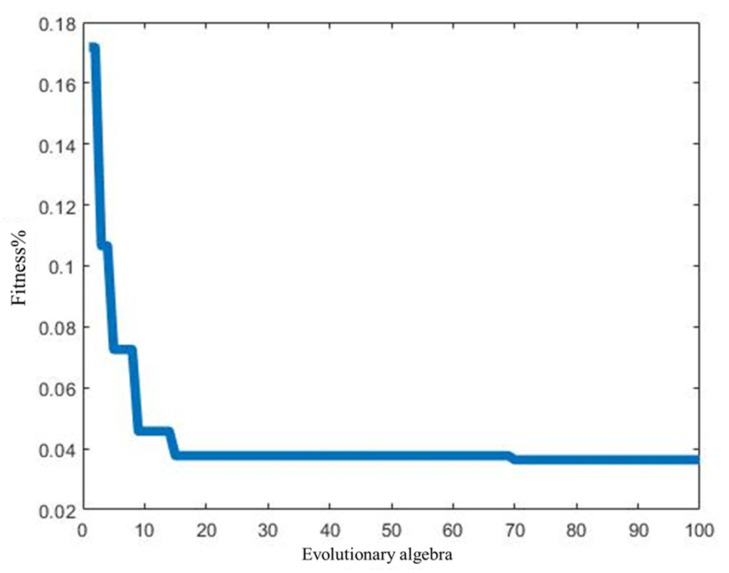
Fitness curve of PSO-LSSVM model.

Using the MATLAB simulation platform to establish the PSO-LSSVM model, the model initialization parameters were set as follows: population size q = 20, maximum number of iterations $t_{\max}$ = 100, learning factors $c_{1}$ = 1.5, $c_{2}$ = 1.7, inertia weight coefficient $\omega =\left[0.4,0.95\right]$, regularization parameter γ∈[0.1,100], and kernel function width coefficient σ∈[0.01,1000]. The 60 normalized data sets were divided into two groups. The first 48 groups are training samples for the model to train and learn on. The last 12 groups are used as test samples for prediction to obtain the fitness curve of the PSO-LSSVM model. As shown in Figure 4.

**Figure 5:**
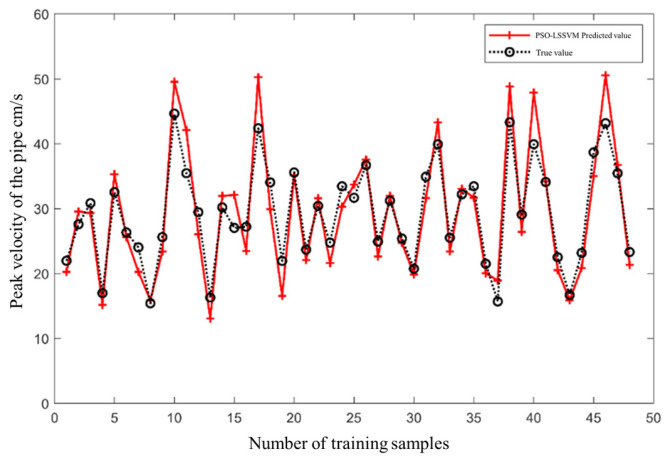
A comparison between the true value and the predicted value of the training sample of the PSO-LSSVM model.

It can be seen from Figure 4 that when the evolutionary algebra reaches 70, the fitness curve has stabilized. The optimal parameter combination at this time is $v_{best}=(21.10,150.94)$. The optimal parameter combination is brought into the model and the training samples are predicted. The comparison between the true value and the predicted value of the training sample is shown in Figure 5. As can be seen from Figure 5, the overall training effect of the PSO-LSSVM model is good. The root mean square error obtained from statistics between the true value of the training sample and its predicted value is RMSE = 0.05, and the correlation coefficient $R^{2}=0.94$, indicating that the regression fitting effect of the model is good.

### 4.2. Comparative Analysis of Forecast Results

**Figure 6:**
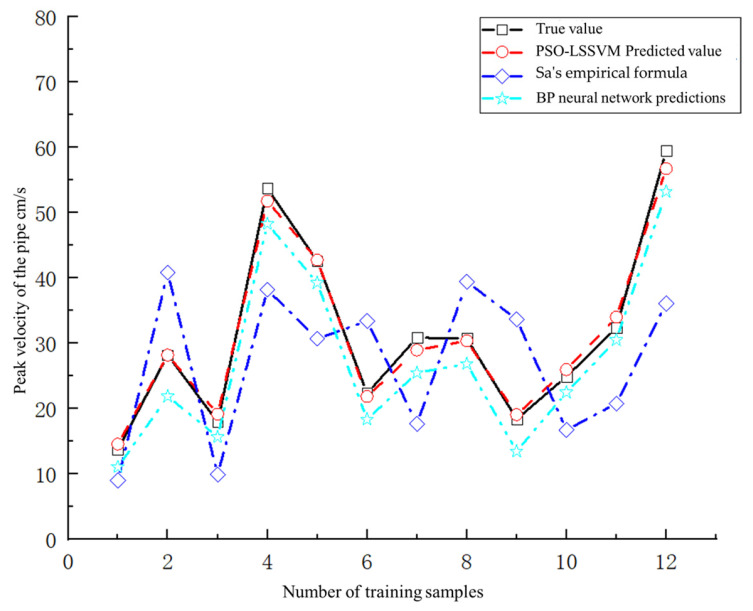
Comparison of prediction results of different models.

The regression fitting of the training samples demonstrates that the PSO-LSSVM model exhibits robust learning capabilities. To verify whether the PSO-LSSVM model is also capable of accurate prediction, 12 sets of test sample data were input for prediction and compared with the BP neural network model and vibration model. Comparative analysis was carried out using the empirical formula and correction formula for rapid prediction. The results comparing the predicted values of the pipeline’s peak vibration velocity by the four models and the actual values are shown in Figure 6.

The comparative analysis chart indicates that the predicted value obtained from the PSO-LSSVM model is the closest to the true value, and the effect is significantly better than the BP neural network model and the empirical formula. In order to further quantify and compare the prediction accuracy of each model, the model evaluation indicators are calculated as follows: fitting correlation coefficient ($R^{2}$), root mean square error (*RMSE*), mean relative error (*MRE*), and Nash coefficient (*NSE*).

In order to avoid the outliers in the data sets leading to a decrease in model prediction accuracy and to make the results more credible, K-fold cross-validation [21] was used to test the data set and model, and *K* = 5. The steps are:

(1) Divide the entire data set into five equal parts;

(2) Take one of them as the test set and, in turn, use the remaining four as the training set to train the model, and calculate the evaluation index of each model prediction result;

(3) The final evaluation index of the model is obtained by averaging the evaluation indexes obtained from the five predictions.

The final statistical results of each evaluation index of the model after K-fold cross-validation are shown in Table 6.

As can be seen from Table 6, when using Sa’s empirical formula to predict the peak vibration velocity of the pipeline $R^{2}$ is 0.88, *RMSE* is 19.68%, and *NSE* is 33.99%, and the model has the largest volatility, *MRE* is 0.78, and it is shown that this formula has the worst prediction ability in this study. The $R^{2}$ of the BP neural network model is 0.88 and the *RMSE* is 0.76. Compared with the empirical formula, its prediction accuracy is greatly improved, indicating that the use of computer network technology and machine learning methods is more suitable for the prediction the physical quantities of blasting engineering with fewer data set samples. The LS-SVM model optimized by the PSO algorithm has the smallest *RMSE* and *MRE*. The model has the highest prediction accuracy and the smallest volatility. The values of $R^{2}$ and *NSE* are the largest. The model has better fitting effects and can more accurately predict blast load effects. It can more accurately predict the peak vibration velocity of buried pipelines under blast load.

## 5. Conclusions

Based on the least squares support vector mechanism theory, a buried pipeline blast vibration prediction model based on PSO-LSSVM was constructed. The prediction results of the PSO-LSSVM model were compared with the prediction results of the BP neural network model and a traditional empirical formula. The main conclusions are as follows:

(1) An LS-SVM model for predicting the blast vibration velocity of buried pipelines was established. The parameters of the LS-SVM model were optimized through the PSO algorithm, and the optimal parameter combination of the LS-SVM model $V_{best}=(21.10,150.94)$ was determined, overcoming the problem of a traditional LS-SVM model’s chosen key parameters being by experience, which leads to low prediction accuracy. The K-fold test method was used to test the vibration velocity model prediction results, which effectively avoids the probability of a reduced model prediction accuracy and improves the reliability of the model’s prediction results.

(2) The PSO-LSSVM model prediction of the peak vibration velocity of the pipeline was $R^{2}$ is 0.92, *RMSE* is 0.29, *MRE* is 0.087, and *NSE* is 0.99. Compared with the BP neural network model and the traditional empirical formula, for a practical problem in blasting engineering practice, such as fewer samples from which to predict the peak vibration velocity of pipelines and a large number of influencing factors due to the higher learning generalization ability and prediction accuracy of PSO-LSSVM model, it can be a better optimized artificial intelligence prediction method.

(3) Due to the limited number of actual blast tests, which resulted in a smaller amount of effective collected data, and the presence of numerous factors influencing blast vibration effects—some of which are not primary factors—future research should expand the sample database to further enhance model accuracy and simplify model calculations.

## Figures and Tables

**Table 1:** Test parameters and control variable ranges.

Test Parameters	Control Variable Scope
Amount of explosive (g)	50, 75, 100, 125, 150, 175, 200
Explosion source burial depth (m)	0.5, 1, 1.5, 2
Pipe internal pressure (MPa)	0, 0.2, 0.4, 0.6, 0.8
Explosion center distance (m)	2.2, 2.7, 3.2

**Table 2:** Steel pipe material parameters.

Density *ρ_s_*/(kg·m^−3^)	Young’s Modulus *E*_S_/GPa	Poisson’s Ratio *μ_s_*	Strength Limit *σ_s_*_b_/MPa	Yield Limit *σ_ss_*/MPa	Elongation *ξ_s_*/%
7850	210	0.30	410	200	25

**Table 3:** Steel pipe geometric parameters.

Pipe Number	Pipe Outer Diameter *D_s_*/mm	Pipe Inner Diameter *d_s_*/mm	Pipe Wall Thickness *δ_s_*/mm	Pipe Length *L_s_*/m
P1	110	101.5	4.24	4.5
P2	160	149.6	4.7	4.5
P3	300	291.2	4.4	4.5

**Table 4:** Data statistics table.

Number of Groups	V/cm/s	*Q*/g	*R*/m	*H_e_*/m	*D*/mm	δ/mm	*H*/m	*P*/MPa
1	20.25	100	2.2	1	110	4.24	0.5	0
2	29.54	150	2.2	0.5	160	4.7	1	0.4
3	29.32	200	2.7	1.5	300	4.4	1.5	0.6
4	15.18	175	3.2	2	160	4.7	1	0.4
5	35.33	200	2.2	1.5	110	4.24	0.5	0.2
6	25.62	150	2.7	1.5	300	4.4	1.5	0.6
7	20.25	100	2.2	1	300	4.4	1.5	0
8	15.89	125	3.2	1	160	4.7	1	0.8
9	23.40	200	2.7	1.5	160	4.7	1	0.4
---	---	---	---	---	---	---	---	---
60	32.39	300	2.7	2	160	4.7	1	0.6

**Table 5:** Characteristic parameters influence factor correlation.

*Q*	*R*	δ	*P*	*D*	*H_e_*	*H*
0.789	0.763	0.703	0.685	0.627	0.618	0.605

**Table 6:** Table of Model Evaluation Metrics.

Formula Type	*MRE*	NSE	$R^{2}$	*RMSE*
PSO-LSSVM Model	0.0869	0.9903	0.9151	0.2954
BP neural network model	0.3476	0.8976	0.8848	0.7601
Sa’s empirical formula	0.7849	0.2014	0.6286	2.8444

## Data Availability

Data are available upon request to the corresponding author.
